# *Ziziphus nummularia* Inhibits Inflammation-Induced Atherogenic Phenotype of Human Aortic Smooth Muscle Cells

**DOI:** 10.1155/2017/4134093

**Published:** 2017-05-15

**Authors:** Manal Fardoun, Tuqa Al-Shehabi, Ahmed El-Yazbi, Khodr Issa, Fouad Zouein, Dina Maaliki, Rabah Iratni, Ali H. Eid

**Affiliations:** ^1^Department of Biology, American University of Beirut, Beirut, Lebanon; ^2^Quality Assurance Department, Mondelēz Bahrain Biscuits W.L.L, Al Hidd, Bahrain; ^3^Department of Pharmacology and Toxicology, American University of Beirut, Beirut, Lebanon; ^4^Department of Pharmacology, Alexandria University, Alexandria, Egypt; ^5^Department of Biology, United Arab Emirates University, Al-Ain, UAE; ^6^Department of Biological and Environmental Sciences, Qatar University, Doha, Qatar

## Abstract

Cardiovascular disease (CVD) continues to be the leading cause of death worldwide. Atherosclerosis is a CVD characterized by plaque formation resulting from inflammation-induced insults to endothelial cells, monocytes, and vascular smooth muscle cells (VSMCs). Despite significant advances, current treatments for atherosclerosis remain insufficient, prompting the search for alternative modalities, including herbal medicine. *Ziziphus nummularia* is an herb commonly used in the amelioration of symptoms associated with many health conditions such as cold, diarrhea, cancer, and diabetes. However, its effect on the inflammation-induced behavior of VSMCs remains unknown. In this study, we sought to determine the effect of the ethanolic extract of *Z. nummularia* (ZNE) on TNF-*α*-induced phenotypic changes of human aortic smooth muscle cells (HASMCs). The treatment of HASMCs with ZNE decreased cell proliferation, adhesion to fibronectin, migration, and invasion. ZNE treatment also caused a concentration- and time-dependent reduction in the TNF-*α*-induced expression of matrix metalloproteases MMP-2 and MMP-9, NF-*κ*B, and cell adhesion molecules ICAM-1 and VCAM-1. Furthermore, ZNE decreased the adhesion of THP-1 monocytes to HASMCs and endothelial cells in a concentration-dependent manner. These data provide evidence for the anti-inflammatory effect of *Ziziphus nummularia*, along with potential implications for its use as an agent that could ameliorate inflammation-induced atherogenic phenotype of VSMCs in atherosclerosis.

## 1. Introduction

Atherosclerosis is one of the leading causes for morbidity and mortality in Western countries [1]. Major adverse cardiovascular events, including stroke and myocardial infarction, are considered as sequelae of atherosclerosis. Atherosclerotic lesions develop gradually as a result of the accumulation of lipids, foam cells, extracellular matrix, and macrophages within the arterial wall [2]. Proinflammatory signals trigger vascular smooth muscle cells (VSMCs) to switch from a quiescent “contractile” phenotype to a synthetic one [3]. VSMCs release matrix metalloproteinases (MMPs), which are enzymes that allow them to detach from extracellular matrix and migrate to the tunica intima. VSMCs then contribute to the growth of atherosclerotic lesions, due to their ability to proliferate and synthesize extracellular matrix and secrete adhesion molecules that recruit and stabilize inflammatory cells, and might even take up lipids and contribute to foam cell formation [4]. Thus, proliferation and migration of VSMCs are key events in atherosclerosis development.

The leaves of *Ziziphus nummularia* (Burm.f.) Wight & Arn (family Rhamnaceae) have been widely used in traditional medicine for the treatment of cold, cutaneous diseases, and pain [5, 6]. Phytochemical studies of *Z. nummularia* extracts revealed the presence of tannins, flavonoids, steroids, glycosides, and alkaloids [7]. The anti-inflammatory activity of *Z. nummularia* has been illustrated by its ability to promote wound healing [8], reduce inflammatory parameters in multiple in vivo models of inflammation, and reduce nitric oxide and TNF-*α* production from activated macrophages in vitro [9]. Importantly, an extract of *Z. nummularia* has been shown to possess DPPH radical scavenging activity [10]. A recent study has also found that the levels of LDL, TG, and VLDL were significantly reduced in diabetic model rats treated with the aqueous and ethanolic extract of *Ziziphus nummularia* [11]. These observations suggest that *Z. nummularia* may have a beneficial effect that could ameliorate atherosclerosis, a disease that could be precipitated and exacerbated by inflammation.

In this study, we investigated the effects of the ethanolic extract of *Z. nummularia* leaves (ZNE) on the proliferation, migration, invasion, and adhesion of HASMCs. Our results demonstrated that ZNE decreased these parameters in HASMCs in a concentration-dependent manner. In addition, the treatment of HASMCs with ZNE inhibited TNF-*α*-induced MMP-2 and MMP-9 production and increased monocyte adhesion.

## 2. Materials and Methods

### 2.1. Cell Culture

Human aortic smooth muscle cells (HASMCs) (Cell Applications, USA) were cultured in DMEM/F12 (50 : 50) and supplemented with L-glutamine, antibiotic/antimycotic cocktail, and 10% fetal bovine serum (FBS). Cells were made quiescent by incubating them in serum-free medium for at least 24 hours prior to any treatment. HUVECs or THP-1 cells (both from American Type Culture Collection, USA) were cultured in RPMI-1640 and supplemented with 10% FBS and 1% penicillin/streptomycin. Cells were maintained in a humidified incubator at 37°C with 5% CO_2_ atmosphere. Culture media, FBS, L-glutamine, antibiotic/antimycotic, and penicillin/streptomycin were obtained from ThermoFisher Scientific, USA.

### 2.2. *Ziziphus nummularia* Extract (ZNE)

The leaves of *Ziziphus nummularia* were rinsed and air dried in the dark at room temperature. They were then ground into powder and were then suspended in 70% ethanol. The mixture was kept in the dark for 72 hours at 4°C, then filtered. The filtrate was evaporated to dryness using a rotary evaporator at room temperature. The obtained residue was kept at −20°C until further use.

### 2.3. Cell Counting by Trypan Blue

HASMCs were seeded in 24-well plates and allowed to grow until they reached 30–40% confluency. Cells were then incubated in 0.5% FBS-containing medium for 24 hours. Following this, regular 10% FBS-containing medium was then added along with increasing concentrations of ZNE for 24, 48, or 72 hours. After aspirating media, wells were washed with PBS, and cells were trypsinized followed by counting using a hemocytometer.

### 2.4. Viability Assay

HASMCs were seeded in 96-well plates and allowed to grow until they reached 30–40% confluency. Cells were then incubated in 0.5% FBS-containing medium for 24 hours. Following this, regular 10% FBS-containing medium was then added along with increasing concentrations of ZNE for 24, 48, or 72 hours. Viability was then determined using CellTiter-Glo assay according to the manufacturer's protocol (Promega, USA). Briefly, equal volumes of CellTiter-Glo reagent and medium were added to the wells. Plates were mixed by an orbital shaker and incubated at room temperature for 10 minutes to stabilize luminescence signals. The GloMax® 20/20 Luminometer (Promega, USA) was used to assess cell viability, in which luminescence is directly proportional to the number of viable cells.

### 2.5. Wound Healing or Scratch Assay

HASMCs were seeded in 6-well plates and grown until confluence. They were then incubated in quiescence media (containing 0.5% FBS) for 24 hours. Using a yellow tip (20–200 *μ*l), a scratch was then made. The culture medium was then aspirated, and plates were washed twice with PBS to remove cellular debris. Fresh medium was then added, and cells were incubated in the absence or presence of ZNE. Photomicrographs were taken at baseline (0 hours) and then at 24 hours using the Olympus IX71 inverted microscope. Olympus cellSens® digital imaging software was used to measure the width of the scratch indicative of cell migration.

### 2.6. Transwell Migration Assay

Cells were seeded onto the upper chamber of a transwell tissue culture plate in serum-free medium, in the absence or presence of ZNE. Regular growth medium (containing 10% FBS) was added in the lower chamber to act as a chemoattractant. After incubation, cells were washed with PBS and fixed. Cells on the upper side of the transwell insert were removed by a cotton-tipped applicator. Cells that have migrated to the lower side of the insert were stained with 1% crystal violet for 10 minutes, followed by washing with PBS. Cells from at least 5 different random fields were counted under the Olympus IX71 inverted microscope.

### 2.7. Invasion Assay

Invasion assay was performed using Matrigel-coated BD BioCoat™ filter inserts (8 *μ*m pore size, BD Biosciences) as per the manufacturer's instructions. Briefly, cells in serum-free media were seeded onto the upper transwell chamber in the absence or presence of ZNE. The lower chamber was loaded with regular growth medium (10% FBS) to act as a chemotactic attractant. Cells were then incubated at 37°C for 24 hours. Cells that did not invade the Matrigel were removed from the upper surface with a cotton-tipped applicator; those that have invaded were stained with crystal violet and visualized under the inverted microscope (Olympus IX71).

### 2.8. Adhesion Assay

Cells were seeded onto fibronectin-coated 96-well plates. Cells were allowed to adhere for 1 hour at 37°C. Nonadherent cells were removed by gentle washing (three times) of the wells using prewarmed PBS. Adherent cells were pictured and counted.

### 2.9. Measurement of MMP-2 and MMP-9

HASMCs were grown until they reached near confluence. MMP production was induced by exposure to FBS or TNF-*α* (10 ng/ml) (R&D Systems, USA). For TNF-*α*, cells were incubated in serum-free media and pretreated with increasing concentrations of ZNE (0, 50, 100,150, and 200 *μ*g/ml) for 30 minutes prior to exposure to TNF-*α*. In the case of FBS, cells were exposed to ZNE 24 or 48 hours in FBS-supplemented media. In all experiments, conditioned media were collected and subjected to an ELISA for MMP-2 and MMP-9 as per the manufacturer's protocol (R&D Systems, USA). Experiments were conducted in triplicates, and data are presented as percent of control (vehicle).

### 2.10. Monocyte Adhesion Assay

The adhesion of THP-1 cells to human umbilical vein endothelial cells (HUVECs) and HASMCs was studied as previously described [12]. Cells were seeded onto collagen-coated plates until confluence, at which point they were treated with increasing concentrations of ZNE for 1 hour. Cells were then incubated with TNF-*α* (10 ng/ml) for 3 hours. THP-1 cells (labelled with 2′,7′-bis-(2-carboxyethyl)-5-(and-6)-carboxyfluorescein; ThermoFisher Scientific) were added atop the confluent cells and allowed to adhere for 30 minutes. Wells were then washed three times to remove the nonadherent THP-1 cells, and the remaining (adhering) THP-1 cells were examined under a microscope. Fluorescence intensity was measured using a microplate reader at excitation and emission wavelengths of 485 and 528 nm, respectively. The adhesion of THP-1 cells in ZNE-treated wells was calculated by expressing the fluorescent intensity relative to that observed in wells treated only with TNF-*α*.

### 2.11. Determination of Expression of Adhesion Molecules by ELISA

The levels of adhesion molecules expressed on the surface of HUVECs was determined by ELISA (Abcam) as previously described with minor modifications [13]. Highly confluent cells were pretreated and incubated with increasing concentrations of ZNE for 2 hours. Cells were then incubated with TNF-*α* (10 ng/ml) for 8 additional hours. Cells were then washed with PBS and fixed with 4% paraformaldehyde for 30 minutes at 4°C. After washing with PBS, wells were blocked with bovine serum albumin (1.0% in PBS), followed by incubation with ICAM-1, VCAM-1, or isotype-matched control antibodies (diluted in blocking buffer; Abcam) overnight at 4°C. Wells were then washed with PBS and incubated with alkaline phosphatase-conjugated secondary antibody. Wells were then washed with PBS and exposed to the peroxidase substrate p-nitrophenyl phosphate (Sigma Aldrich, USA) (1 mg/ml in 0.1 M glycine buffer, pH 10.4, containing 1 mM MgCl_2_, and 1 mM ZnCl_2_). Absorbance was measured at 405 nm, and the values of the isotype-matched control wells were taken as blank.

### 2.12. Quantitative Real-Time Polymerase Chain Reaction (RT-PCR)

HUVECs were cultured till confluency. They were treated with increasing concertation of ZNE for 30 minutes followed by the addition of TNF-*α* (10 ng/ml). After (time x), cells were lysed and total RNA was extracted using TRIzol reagent (Invitrogen) according to the manufacturer's instructions. RNA was then converted into cDNA by reverse transcription, and quantitative real-time PCR was performed using the GoTaq® 2-Step System (Promega). mRNA of ICAM-1 and VCAM-1 was reverse transcribed using the following primers: VCAM-1—forward CCCTTGACCGGCTGGAGATT and reverse CTGGGGGCAACATTGACATAAAGTG; ICAM-1—forward CTGTCACTCGAGATCTTGAGG and reverse CCTGCAGTGCCCATTATGA; and GAPDH—forward ACCCAGAAGACTGTGGATGG and reverse TTCTAGACGGCAGGTCAGGT. Changes in expression levels were calculated with the 2^−ΔΔCT^ method.

### 2.13. Luciferase Assay

HUVECs were transfected with NF-*κ*B-driven promoter luciferase along with Renilla luciferase vector as an internal control. Transfection and luciferase protocols are discussed previously [14, 15]. Cells were treated with TNF-*α* alone or with increasing concentrations of ZNE prior to TNF-*α* treatment. NF-*κ*B expression was quantified by the relative promoter unit and expressed as percentage of expression in cells treated with vehicle alone.

## 3. Statistical Analysis

Statistical analysis of the data was performed by one-way or two-way ANOVA followed by Dunnett's, Tukey's, or Sidak's tests for multiple comparisons as appropriate using GraphPad Prism Software (GraphPad Software Inc., San Diego, CA). Data were presented as mean ± SEM. A *p* value of less than 0.05 was considered as significant.

## 4. Results

### 4.1. ZNE Inhibits FBS-Induced HASMC Proliferation, Migration, and Adhesion to Fibronectin

**Figure 1:**
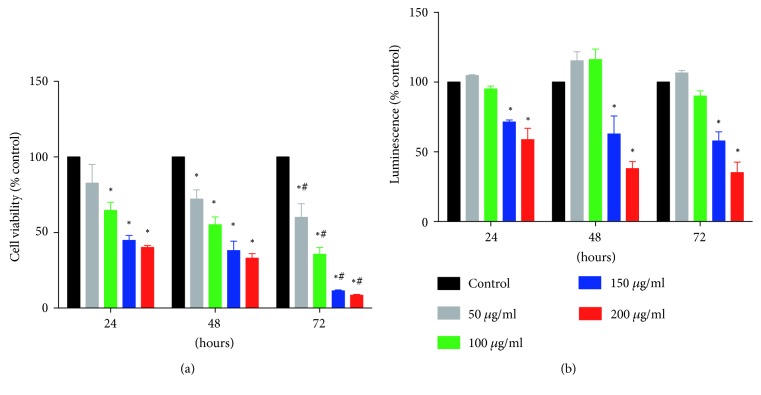
ZNE decreases VSMC proliferation in a concentration- and time-dependent manner as demonstrated by trypan blue cell count at the indicated time intervals (a) and the overall metabolic activity measured by CellTiter-Glo luminescent cell viability assay (b). Viability values are calculated as % of the corresponding vehicle control value and represented as mean ± SEM of three replicates. Statistical significance was tested using 2-way ANOVA followed by Dunnett's post-hoc test. ∗ denotes *P* < 0.05 compared to that of the vehicle control, while # indicates *P* < 0.05 compared to the effect of the same concentration of ZNE at 24 hours.

The inhibitory effects of ZNE on FBS-induced HASMC proliferation were studied using two different assays. Overall, ZNE treatment showed a concentration- and time-dependent inhibition of the FBS-induced HASMC proliferation (Figures 1(a) and 1(b)). In trypan blue exclusion assay (Figure 1(a)), ZNE concentrations of 100, 150, and 200 *μ*g/ml significantly reduced the viable cell number at 24, 48, and 72 hours. The lowest concentration (50 *μ*g/ml), however, causes a significant reduction in cell number only after 48 and 72 hours of ZNE treatment. In the CellTiter assay that measures metabolic activity (which corresponds to viability), only the ZNE concentrations of 150 and 200 *μ*g/ml significantly reduced the viable cell number at 24, 48, and 72 hours (Figure 1(b)).

**Figure 2:**
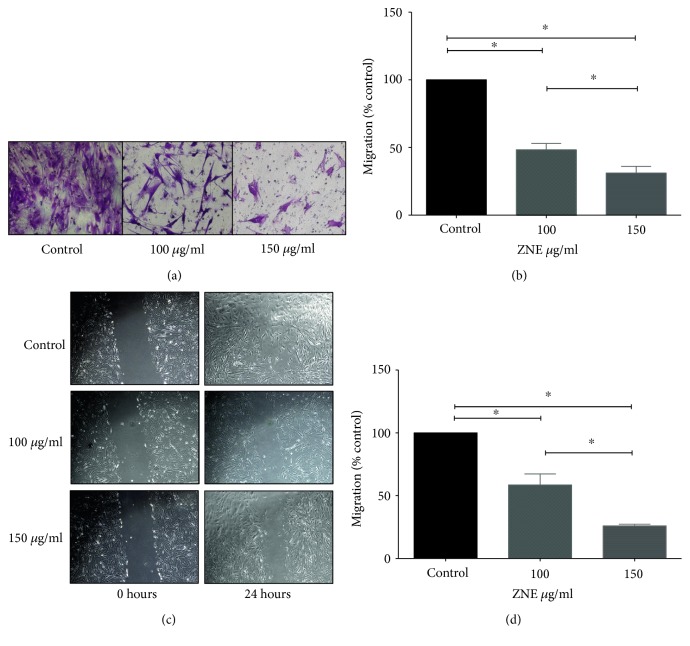
ZNE decreases VSMC migration in a concentration-dependent manner. (a) Representative photomicrographs of the effect of 100 and 150 *μ*g/ml of ZNE on VSMC migrating to the lower chamber in transwell migration assay. (b) Mean ± SEM of % control values for VSMC migration to the lower chamber (*n* = 3 replicates). (c) Representative photomicrographs of the effect of 100 and 150 *μ*g/ml of ZNE on VSMC scratch wound healing at 24 hours postscratch. (d) Mean ± SEM of % control values of the residual scratch wound area (*n* = 3 replicates). Statistical significance was tested with ANOVA followed by Tuckey's post-hoc test. ∗ denotes *P* < 0.05 on the indicated comparisons.

The ZNE effect on FBS-induced migration of HASMCs was assessed using both scratch and transwell migration assays (Figure 2). ZNE at 100 and 150 *μ*g/ml concentrations significantly decreased FBS-induced HASMC migration from the upper to lower chambers (Figures 2(a) and 2(b)). Similarly, ZNE treatment at both concentrations delayed scratch wound healing compared to vehicle-treated cells (Figures 2(c) and 2(d)).

**Figure 3:**
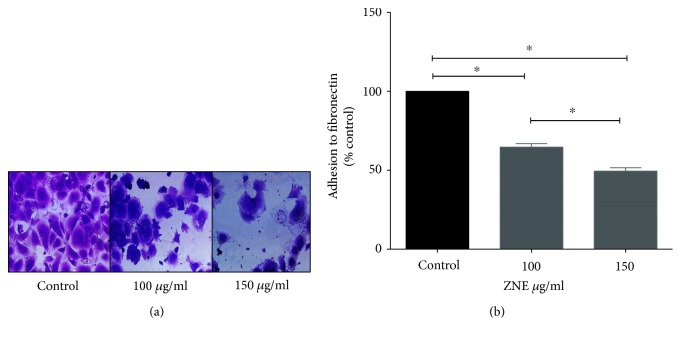
ZNE decreases VSMC adhesion to fibronectin in a concentration-dependent manner. (a) Representative photomicrographs of the effect of 100 and 150 *μ*g/ml of ZNE on VSMC adhesion to fibronectin. (b) Invasion data summary represented as mean ± SEM of % corresponding control values for VSMC adhesion (*n* = 3 replicates). Statistical significance was tested with ANOVA followed by Tuckey's post-hoc test. ∗ denotes *P* < 0.05 on the indicated comparisons.

Because the adhesion of VSMCs to extracellular matrix is important for cell proliferation, migration, and invasion, we sought to determine the effect of ZNE on the adhesion of VSMCs to fibronectin. Our results show that the adhesion of HASMCs to extracellular matrix, assessed using fibronectin-coated plates, showed that pretreatment with ZNE significantly inhibited adhesion to fibronectin in a concentration-dependent manner (Figure 3).

### 4.2. ZNE Inhibits TNF-*α*- and FBS-Evoked Invasion and MMP-2 and MMP-9 Secretion in HASMCs

**Figure 4:**
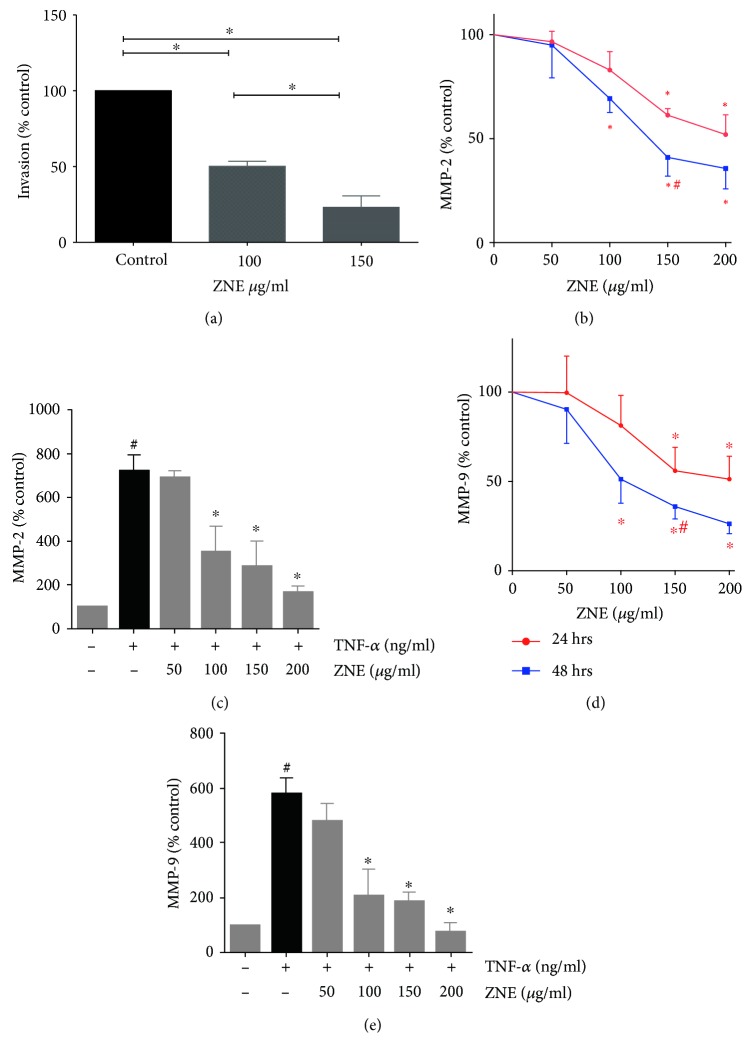
The effect of ZNE on VSMC invasion. (a) The effect of 100 and 150 *μ*g/ml of ZNE on VSMC invasion. Values represented are mean ± SEM of % corresponding control values for VSMC invasion into Matrigel (*n* = 3 replicates). Treatment with increasing concentrations of ZNE is associated with graded inhibition of both spontaneous (b, d) and TNF-*α*-evoked (c, e) MMP-2 and MMP-9 levels in VSMCs. Data represented are mean ± SEM of % MMP level in the corresponding vehicle control treatment (*n* = 3 replicates). For (b, d), statistical significance was tested with 2-way ANOVA followed by Dunnett's post-hoc test for the effect of concentration at the same exposure time (∗ denotes *P* < 0.05 compared to that of the vehicle control) and Sidak's post-hoc test for the effect of time at the same concentration (# denotes *P* < 0.05 compared to that of the corresponding concentration at 24 hours). For (c, e), statistical significance was tested with ANOVA followed by Tuckey's post-hoc test. ∗ denotes *P* < 0.05 compared to that of the MMP level evoked by TNF-*α* in the absence of ZNE, while # denotes *P* < 0.05 compared to that of the MMP level of the vehicle control.

Inflammatory cytokines, including TNF-*α*, are known to trigger invasion of HASMCs mediated by matrix metalloproteinases [16, 17]. Our results here show that treatment with ZNE (100 and 150 *μ*g/ml) inhibited HASMC invasion of the Matrigel-coated filter inserts. ZNE significantly reduced invasion by 25% and 50% at concentrations 100 and 150 *μ*g/ml, respectively (Figure 4(a)). Levels of MMP-2 and MMP-9 secreted in the conditioned media of HASMCs exposed to TNF-*α* in the absence and presence of ZNE were assessed with ELISA. Similar experiments were conducted in VSMCs exposed to different concentrations of ZNE in FBS-supplemented media. In accordance with the results of cell invasion assays, ZNE, at 100, 150, and 200 *μ*g/ml concentrations, significantly reduced MMP-2 (Figures 4(b) and 4(c)) and MMP-9 (Figures 4(d) and 4(e)) secretion in a time- and concentration-dependent manner.

### 4.3. ZNE Inhibits TNF-*α*-Induced Adhesion of Monocytes to Endothelial and Smooth Muscle Cells

**Figure 5:**
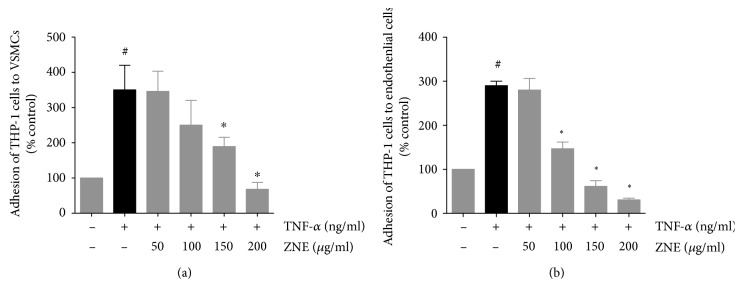
Treatment with increasing concentrations of ZNE leads to a graded decrease in TNF-*α*-evoked adhesion of THP-1 cells to VSMCs (a) and HUVECs (b). Values represented are mean ± SEM of % THP-1 cell adhesion upon treatment with the corresponding vehicle control (*n* = 3 replicates). Statistical significance was tested with ANOVA followed by Tuckey's post-hoc test. ∗ denotes *P* < 0.05 compared to that of the THP-1 cell adhesion evoked by TNF-*α* in the absence of ZNE, while # denotes *P* < 0.05 compared to that of the cell adhesion seen upon treatment with the vehicle control.

Monocyte adhesion is a hallmark of atherosclerosis. In order to investigate the effect of ZNE on monocyte adhesion to TNF-*α*-activated endothelial or smooth muscle cells, THP-1 cells were incubated with TNF-*α*-treated HUVECs or HASMCs, with or without prior exposure to increasing concentrations of ZNE. In the absence of TNF-*α* stimulation, no or minimal number of monocytes adhered to HASMCs or HUVECs (Figures 5(a) and 5(b), respectively). However, a clear and significant increase in monocyte adhesion was observed following TNF-*α* treatment. Pretreatment with ZNE decreased monocyte adhesion on the TNF-*α*-activated cells in a concentration-dependent manner (Figure 5()).

### 4.4. ZNE Inhibited TNF-*α*-Induced ICAM-1 and VCAM-1 Expression in Endothelial Cells

**Figure 6:**
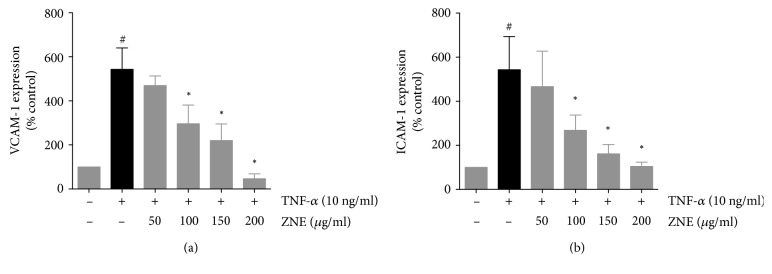
Gradual reduction in TNF-*α*-evoked adhesion molecule expression ((a), VCAM-1; (b), ICAM-1) on HUVECs detected by cell-surface ELISA upon treatment with increased concentrations of ZNE. Values represented are mean ± SEM of % adhesion molecule expression observed upon treatment with the corresponding vehicle control (*n* = 3 replicates). Statistical significance was tested with ANOVA followed by Tuckey's post-hoc test. ∗ denotes *P* < 0.05 compared to that of the adhesion molecule expression evoked by TNF-*α* in the absence of ZNE, while # denotes *P* < 0.05 compared to that of the expression observed upon treatment with the vehicle control.

The interaction of endothelial cells and monocytes in atherosclerosis is mediated through adhesion molecules, among which are VCAM-1 [18] and ICAM-1 [19]. The effect of ZNE on the expression of ICAM-1 and VCAM-1 in TNF-*α*-stimulated HUVECs was examined on the protein and mRNA levels. TNF-*α* treatment of HUVECs showed ~3-fold increase in VCAM-1 (Figure 6(a)) and ICAM-1 (Figure 6(b)) protein expression levels compared to untreated controls in cell surface ELISA assays. However, pretreatment with ZNE decreased the ICAM-1 and the VCAM-1 protein expression in the TNF-*α*-activated HUVECs in a concentration-dependent manner to a level that is similar to the control values (Figure 6). A similar effect was observed in RT-PCR assays on the ICAM-1 and VCAM-1 transcripts (data not shown).

### 4.5. ZNE Inhibits TNF-*α*-Induced Expression of NF-*κ*B in a Concentration-Dependent Manner

**Figure 7:**
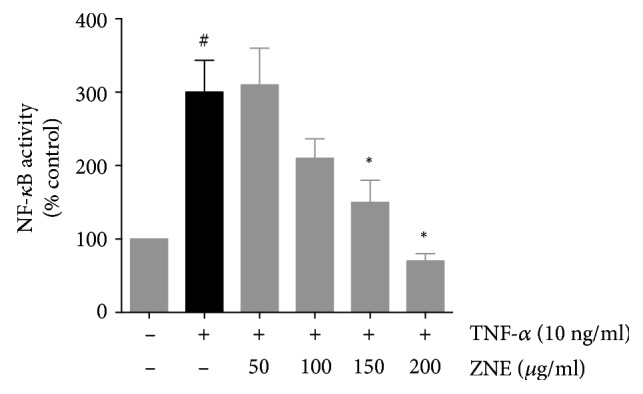
TNF-*α*-evoked NF-*κ*B expression in HUVECs transfected with a luciferase-expressing NF-*κ*B promotor is decreased by ZNE treatment in a concentration-dependent manner. Values represented are mean ± SEM of % promotor units recorded upon treatment with the corresponding vehicle control (*n* = 3 replicates). Statistical significance was tested with ANOVA followed by Tuckey's post-hoc test. ∗ denotes *P* < 0.05 compared to that of the expression evoked by TNF-*α* in the absence of ZNE, while # denotes *P* < 0.05 compared to that of the expression observed upon treatment with the vehicle control.

Several atherogenic stimuli converge at the NF-*κ*B signaling pathway. TNF-*α* activates NF-*κ*B transcription factor translocation to the nucleus with subsequent activation of the transcription of many genes, including adhesion molecules [20]. Whether ZNE affected the transcription of adhesion molecules through NF-*κ*B pathway was tested using luciferase assay. As shown in Figure 7, HUVECs treated with TNF-*α* showed considerable increase in NF-*κ*B expression. However, this was reversed by preexposure of TNF-*α*-activated HUVECs to increasing concentrations of ZNE in a concentration-dependent manner.

## 5. Discussion

Pathological alternation of VSMCs is a main player in atheroma progression [21]. VSMCs lose their ability to regulate vasocontraction and switch to a synthetic phenotype in response to inflammatory signals [22]. Locally produced growth factors and cytokines stimulate the migration of VSMCs into the intima and the subsequent proliferation. These migrated VSMCs synthesize ECM proteins that can stabilize the atherosclerotic plaque [22]. Consequently, inhibiting the proliferation and migration of VSMCs would confer a beneficial effect in atherosclerosis prevention. Moreover, VSMCs secrete MMP-2 and MMP-9 enzymes that can degrade the extracellular matrix enabling leukocyte influx, thinning of the fibrous cap and angiogenesis [23]. In vivo studies showed that MMP-9 regulates both VSMC migration and invasion [24].

Here, we showed that the ethanolic extract of *Z. nummularia* inhibited key events that are integral to the process of atherosclerosis. Indeed, ZNE inhibited FBS-induced proliferation as well as the migration of human aortic smooth muscle cells. Adhesion to fibronectin, an extracellular protein, was also inhibited by ZNE. Importantly, these inhibited activities were concomitant with decreased production of metalloproteases (MMP-2 and MMP-9) as well as diminished invasive capacity of these cells.

*Ziziphus nummularia*, family Rhamnaceae, is native to India, Pakistan, and Iran but is also widely found in the deserts of the Arabian Peninsula. It is widely used in folk medicine of various countries (Iran, India, Pakistan, and others) as a treatment for a wide spectrum of ailments such as cold, cough, throat inflammation, and diabetes as well as a sedative, aphrodisiac, and an antiseptic [25, 26]. A recent study identified a new terpene isolated from *Z. nummularia* that appears to possess potent anticancer activity, both in vitro and in vivo [26]. Relevantly, the leaves of *Z. nummularia* have been recently shown to elicit an anti-inflammatory effect [6]. It is this anti-inflammatory capacity that is attractive and desirable for the potential utilization of this herb in the treatment or management of atherosclerosis. Here, we studied the effects of *Ziziphus nummularia* on TNF-*α*-activated HUVECs and HASMCs, an inflammation model mimicking atherogenesis. We found that the ethanolic extract of this plant significantly inhibited the migration and invasion of VSMCs as wells as the adhesion of monocytes on endothelial and smooth muscle cells. These results provide indications for a potential antiatherogenic effect of *Ziziphus nummularia* as a potential herbal supplement, provided that further studies support its safety.

To the best of our knowledge, this is the first study that investigated the atheromodulatory effects of *Ziziphus nummularia.* However, other *Ziziphus* species have been reported to possess antiatherosclerotic properties. *Zizyphusjujuba* contains a variety of triterpenoids that inhibit foam cell formation in macrophages [27]. Also, polyphenols extracted from *Zizyphus jujuba* peels have the potential to prevent isoproterenol-induced myocardial injury [28]. It was also shown that *Z. jujuba* extract can block vascular angiogenesis by inhibiting endothelial MMP-2 production [29]. *Ziziphus oenoplia* is another member of Rhamnaceae family that is suggested to possess antiatherosclerotic properties. The ethanolic extract of *Ziziphus oenoplia* demonstrated hypolipidemic activity in vivo [30]. Moreover, *Ziziphus spina-christi* leaf powder has significantly inhibited hyperlipidemia in high-cholesterol-fed rats [31].

The leaves of *Ziziphus nummularia* are rich in tannins, flavonoids, steroids, glycosides, and alkaloids [7]. Interestingly, tannins were demonstrated to inhibit proliferation and migration of HASMCs [32, 33]. Being a constituent of *Ziziphus nummularia* extract, tannins may be imparting these antiproliferative and antimigratory effects; however, this remained to be established. Importantly, the decrease in HASMC migration and invasion was coupled to a reduced MMP-9 and MMP-2 expression, providing a potential mechanism to underlie the effect of ZNE. To the best of our knowledge, this is the first report to show reduced cellular migration and invasion by any species of the *Ziziphus* genus; hence, this is the significance of our findings.

In the context of atherosclerosis, the binding of VSMCs to fibronectin is of marked importance since fibronectin is known to modulate arterial smooth muscle cell dedifferentiation from a contractile phenotype to a synthetic one [34]. Indeed, it has long been established that fibronectin plays key roles in the adhesion, proliferation, differentiation, and migration of different cell types [35]. The main fibronectin receptor that is abundantly expressed on the surface of VSMCs is *α*5*β*1 [36]. Importantly, the upregulation of *α*5*β*1 integrin in the neointimal lumen is thought to critically augment VSMCs adhesion to fibronectin during inflammation [36]. With a suggested role in fibrin adhesion and fibrin clot formation [37], *α*5*β*1 could contribute to luminal narrowing of the vessel because of the abundancy of both fibrin and fibronectin in the atherosclerotic lesion [38]. Our results showed that the treatment of HASMCs with ZNE significantly inhibited their fibronectin adhesive capacity. Whether the effect of ZNE on fibronectin adhesion is through *α*5*β*1 modulation in HASMCs remains to be validated.

Plaque formation is precipitated by various irritants, such as increased lipid levels in dyslipidemia or elevated systemic blood pressure in hypertension. These irritants can act as insults that compromise the endothelial integrity resulting in the accumulation of low-density lipoproteins (LDL) in the sub-endothelial space [39, 40]. The oxidation of these trapped LDL can then lead to a series of events, including an increased expression of adhesion molecules [39, 40]. These molecules then promote the interaction between monocytes and endothelial cells during the early stages of atherosclerosis [41]. Indeed, when monocytes bind to these adhesion molecules, they are redirected into the intima by the proinflammatory milieu. Monocytes get attracted to and accumulate in the subendothelial space where they take up lipids to become foam cells [42]. This leads to plaque formation and thickening of the vessel wall due to accumulation of foam cells, VSMCs, and other cell types [42]. In order to determine if ZNE modulates these adhesion molecules, we utilized TNF-*α* to mimic inflammatory stimuli. Indeed, pretreatment of TNF-*α*-activated HUVECs with ZNE led to a significant reduction of monocyte adhesion. Since the interaction between endothelial cells and monocytes or monocyte-derived cells is mediated through adhesion molecules [39, 40] and the upregulation of these molecules is intimately associated with atherosclerosis-prone aortas [39, 40], we examined the effect of ZNE on the expression of two molecules of this family, namely, ICAM-1 and VCAM-1. These two proteins are known to show increased expression on the intimal side of the atherosclerotic plague endothelia but not the adventitial side [43]. Here, we showed that ZNE decreased the expression of both ICAM-1 and VCAM-1 in TNF-*α*-activated endothelial cells on both RNA and protein levels. Thus, one could postulate that this decrease in monocyte adhesion, likely due to reduced adhesion molecules expression, could help in reversing atherosclerosis at its early stages.

NF-*κ*B is a transcription factor that lies at the crossroads of inflammation and atherogenesis [44]. It regulates proteins that play a key role in the pathogenesis of atherosclerosis. Some of these proteins are ICAM-1, VCAM-1, MMP-2, and MMP-9 [45, 46]. Importantly, the critical roles that NF-*κ*B plays are evident in virtually all stages of atherosclerosis, including vascular remodeling, plaque formation, and fatal plaque rupture [44]. In an unstimulated state, NF-*κ*B activity is suppressed by the inhibitor protein, I*κ*B. Several stimuli, including TNF-*α*, activate NF-*κ*B through a series of signaling cascades leading to I*κ*B phosphorylation, which then allows for NF-*κ*B release and nuclear translocation triggering the transcription of atherogenic genes. Our results here show that ZNE decreased NF-*κ*B expression in TNF-*α*-activated endothelial cells providing a molecular context for the observed antiatherogenic effects of ZNE on VCAM-1, ICAM-1, MMP-2, and MMP-9. Interestingly, recent in silico studies showed that a component of *Ziziphus nummularia* can bind to and inhibit the production of TNF-*α* in activated macrophages [9]. This compound, however, was purified from the root bark of *Z. nummularia*; whether it also exists in the leaves remains to be established. This finding supports the potential antiatherosclerotic effects of *Ziziphus nummularia* and identifies a possible pathway by which it elicits these effects.

## 6. Conclusions

In summary, our results showed that the ethanolic extract of *Ziziphus nummularia* leaves has antiatherogenic properties. By inhibiting the inflammation-induced proliferation, migration, invasion, and fibronectin adhesion of HASMCs, as well as reducing reduced VCAM-1 and ICAM-1 expression in endothelial cells, *Z. nummularia* imparts its antiatherosclerotic effects. Ongoing and future studies will focus on isolating and characterizing the effects of the bioactives of the ethanolic extracts as well as the evaluation of the effect of *Ziziphus nummularia* on the aspects of blood vessel function animal models of vascular inflammation.
